# Segmented in vitro fertilization and frozen embryo transfer in levonorgestrel-releasing intrauterine device treated patients with endometrial cancer

**DOI:** 10.1007/s00404-023-07170-x

**Published:** 2023-09-01

**Authors:** Hongyi Wei, Ningning Pan, Yang Wang, Caihong Ma

**Affiliations:** 1 Center for Reproductive Medicine, Department of Obstetrics and Gynecology, Peking University Third Hospital, Haidian District, No. 49 North Huayuan Road, Beijing, 100191 China; 2 National Clinical Research Center for Obstetrics and Gynecology, Beijing, 100191 China; 3 Department of Obstetrics and Gynecology, Peking University International Hospital, Beijing, 102206 China

**Keywords:** Levonorgestrel, Intrauterine device, Endometrial cancer, Ovarian stimulation, In vitro fertilization

## Abstract

**Purpose:**

To evaluate the efficacy of levonorgestrel-releasing intrauterine device (LNG-IUD) during controlled ovarian stimulation (COS) in patients with early-stage endometrioid endometrial cancer (EEC).

**Methods:**

A retrospective study was conducted on patients with stage IA1 EEC who achieved complete response after fertility-sparing treatment from December 2018 to December 2021, with all the women who underwent COS having LNG-IUDs inserted in their uterine cavity.

**Results:**

16 patients were enrolled who underwent 26 COS cycles and average age was 33.19 ± 4.04 years. 12 patients had 19 subsequent frozen-thawed embryo transfer (FET) cycles. Among the other four patients, no embryos were obtained in 1 patient, 1 patient got pregnancy spontaneously with term delivery after COS, 1 patient relapsed before FET, and 1 patient did not receive embryo transfer for personal reason. Among 19 FET cycles, the clinical pregnancy and live birth rates in each ET cycle were 36.84% (7/19) and 26.32% (5/19), respectively. 7 clinical pregnancies resulted in 2 miscarriages (28.6%), and 5 live births (71.4%). Totally 6 patients achieved 7 live births, and the cumulative live birth rate was 37.5% (6/16). Three (18.75%) out of 16 patients relapsed after COS during the follow-up period (31.31 ± 15.89 months) and two of them were initially diagnosed with moderately differentiated EEC. Time interval from COS to relapse was 6.63,11.67 and 16.23 months, respectively.

**Conclusion:**

The combination of LNG-IUD treatment and segmented IVF may be a viable treatment strategy to improve oncological and reproductive outcomes for patients with early-stage EEC.

## **What does this study add to the clinical work**

**Table:** 

The combination of LNG-IUD treatment and segmented-IVF may be a viable treatment strategy to improve oncological and reproductive outcomes for patients with EEC, especially for those who wish to cryopreserve fertility.	

## Introduction

Endometrial cancer (EC) is one of the most common gynecological malignant tumors. About 7% to 8% of EC occurs in women of childbearing age. The standard method for treating EC is total hysterectomy with bilateral salpingo-oophorectomy, peritoneal cytology, and/or lymph node removal. The introduction of molecular subtyping in endometrial cancer has significantly enriched our understanding of the disease, providing crucial insights for patient classification, accurate prognostic prediction, and the development of personalized treatment approaches [1, 2]. As more than 70% of young patients are nulliparous, these patients may not be willing to undergo such radical treatment due to their wish for childbearing. Thus, progestin-based conservative treatment is applied in these young patients with early-stage endometroid EC (EEC). The initial response rate of early EEC patients after progesterone application is 60–80%. Due to the high risk of recurrence (25–40%) and short disease-free interval (12–28 months) after treatment [3, 4], it is recommended that pregnancy should be carried out as soon as possible after successful endometrial remission, and if necessary, assisted reproductive technology (ART) should be actively accepted to improve the pregnancy rate.

Although previous studies have confirmed the efficacy of ART in patients with atypical endometrial hyperplasia and endometrial cancer for pregnancy [5, 6], the pregnancy and live birth rates of these patients remain low. Gallos et al. reported that the live birth rates for atypical endometrial hyperplasia (AEH) and EC patients were 26.3% and 28% respectively, and the pregnancy rate with ART was higher than that of natural conception (39.4% vs 14.9%) [5]. As compared to AEH, patients with EEC had a lower live birth rate and a higher relapse rate during IVF according to previous studies [7, 8]. This could be due to a variety of factors including disease recurrence, damaged endometrium, diminished ovarian reserve, and obesity. Currently, to protect the endometrium from high levels of estrogen that may promote the progression of EEC, levonorgestrel-releasing intrauterine device (LNG-IUD) is applied during controlled ovarian stimulation (COS). LNG-IUD (Mirena®, Bayer Global, Leverkusen, Germany) was originally used for contraception in the mid-1970s. It delivers a concentrated dose of levonorgestrel (20 µg/day) directly to the endometrium while minimizing the systemic side effects. A study of female patients pursuing either social oocyte cryopreservation or oocyte donation showed that the use of LNG-IUDs during COS had no apparent negative impact on cycle performance, including total oocytes, mature oocytes, clinical pregnancy rate, and live birth rate [9]. Limited data is available on the effectiveness and recurrence risk of LNG-IUDs during COS in EEC patients. Kim et.al reported a relapse rate in EEC after COS of 27.27%(6/22) [10]. Our previous study showed a relapse rate of 50% in EEC patients after COS [8]. Yin et.al reported a total recurrence rate of 23.9% in 67 patients with EEC/AEH after COS and compared with patients who did not use LNG-IUD during COS, the recurrence rate (12.1% vs. 35.3%) was lower in the group that used LNG-IUD, whereas the number of EEC patients who had relapsed is not specifically mentioned. The clinical pregnancy and live birth rates were similar between the LNG-IUD and control groups [11].

In this study, we report the oncologic and pregnancy outcomes of 16 women diagnosed with EEC who underwent COS with LNG-IUD after fertility-sparing treatments in one tertiary hospital.

## Materials and methods

### Study design and participants

We performed a retrospective analysis of a prospectively managed database of patients with EEC who achieved complete response after fertility-sparing treatment who sought in vitro fertilization and embryo transfer (IVF-ET) at the Reproductive Center of Peking University Third Hospital from December 2018 to December 2021. This study obtained Institutional Review Board approval (No. M2022572). Histological diagnosis was achieved by obtaining samples by hysteroscopy and D&C. Furthermore, all patients underwent a thorough evaluation, including pelvic examination, ultrasonography, as well as abdominal and pelvic computed tomography or magnetic resonance imaging. The inclusion criteria: (1) clinical stage I A1 (FIGO 2023), histologically proven well or moderately differentiated EEC; MRI/CT confirmed no infiltration of the myometrium and pelvic/aortic node involvement; (2) conservative therapy with megestrol acetate (MA) or medroxyprogesterone acetate (MPA), LNG-IUD combined with GnRH agonist or oral progesterone, and pathological complete response (CR) were achieved; (3) LNG-IUD as a maintenance treatment during COS; and (4) Underwent standard COS protocols and IVF/FET cycles. Patients with incomplete medical records were excluded from the analysis. Clinical and IVF/FET characteristic data, pregnancy outcomes and endometrial disease recurrence data were extracted from both paper and electronic medical records. Patients were followed until April 1st, 2023.

### IVF/FET treatment

The selection of the optimal ovarian stimulation regimen for each individual patient was made by experienced doctors based on a thorough evaluation of various factors such as their age, hormonal levels, body mass index (BMI), ovarian, compliance with medications, and financial constraints. GnRH agonist and antagonist protocols were utilized, along with recombinant FSH or urinary human menopausal gonadotropin in the COS process. The initial dose of Gonadotropin (Gn) was determined based on the patient's ovarian reserve (150–300 U/d) and adjusted as necessary during COS. The GnRH agonist long protocol involved administering 0.1 mg of short-acting GnRH agonist during the luteal phase of the previous menstrual cycle, followed by starting Gn 14 days later until the trigger day. Once serum E_2_ > 1500 pmol/L letrozole might be added at a dosage of 2.5 or 5.0 mg/day until the trigger day. In the GnRH antagonist protocol, Gn was initiated on the second or third day of the menstrual cycle, and when the leading follicle diameter reached 12–14 mm, a daily injection of 0.25 mg of GnRH antagonist was introduced until the trigger day. In the letrozole/antagonist protocol, patients orally received letrozole at a dosage of 2.5 or 5.0 mg/day from the second day of the menstrual cycle for five days within the GnRH antagonist protocol. Ovulation trigger was achieved by administering human chorionic gonadotropin (hCG) (6500 IU) or GnRH agonist (0.2 mg) combined with hCG 2000 IU when at least two follicles reached a diameter greater than 18 mm. Oocyte retrieval was performed 34 to 38 h later. Oocytes were fertilized using conventional IVF or Intracytoplasmic sperm injection (ICSI). The development and quality of embryos were assessed on day 3 by the percentage of fragmentation and quality of cytoplasm. Blastocyst morphology was evaluated on day 5 or 6 using the Gardner grading system [12]. All usable embryos were cryopreserved by vitrification. LNG-IUDs were removed one or two months before the initiation of FET. Subsequent frozen-thawed transfers were performed on either day 3 or day 5, utilizing natural, induced menstrual cycles, or artificial cycles. Each embryo transfer involved the implantation of one or two embryos per patient. After embryo transfer, regular luteal support was provided with oral, vaginal, or intramuscular injections of progesterone per day from the day of ET to the 10th week of gestation.

### Definition and outcome

Complete response was defined as no cancerous or hyperplastic lesions present in the endometrium confirmed by pathology. The time to CR referred to the duration between the initiation of conservative treatment and the achievement of CR. The time to COS was defined as the duration between achieving CR and the beginning of the COS cycle. The time to first FET was defined as the interval between the date of the last oocyte retrieval and the day of first embryo thawing and transfer. The time to live birth referred to the duration between the initiation of COS and the first live birth. The follow-up time was defined from CR to the last follow-up.

The primary outcome was disease relapse after COS. The secondary endpoints were clinical pregnancy and live birth. Clinical pregnancy was diagnosed by ultrasonographic visualization of the intrauterine gestational sac after embryo transfer. Live birth was defined as the delivery of any viable infant after 24 weeks of gestation. The cumulative live birth rate of the study cohort was defined as the number of women who obtained a live birth divided by the number of women involved in the study.

### Statistical analysis

The data were analyzed by SPSS version 26 (SPSS Inc., Chicago, IL, USA). Data are presented as mean ± SD for continuous variables with normal distribution. Non-normally distributed continuous data are presented as median (range). Categorical data are presented as number (percentage).

## Results

**Fig. 1:**
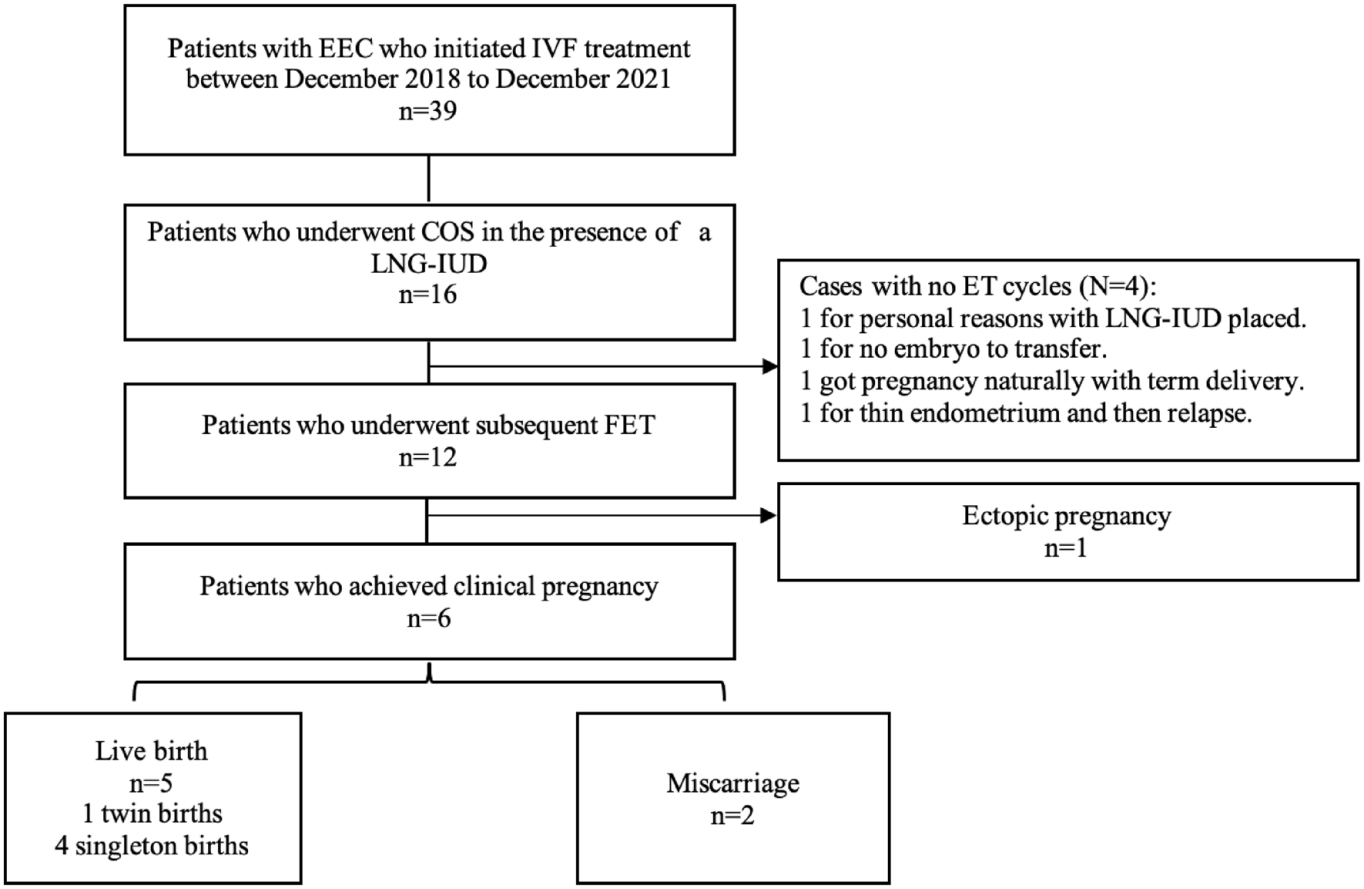
Reproductive outcomes of frozen embryo transfers

As shown in Table 1 and Fig. 1, a total of sixteen patients treated with LNG-IUD while undergoing COS met the inclusion criteria. As shown in Table 2, the mean age of the patients was 33.19 ± 4.04 years (range, 25–39 years), and their average BMI was 28.08 ± 4.16 kg/m^2^ (range, 21.3–37.9 kg/m^2^). The mean number of D&C was 5 ± 0.97 (range, 4–7), and all patients responded to treatment with a mean time to CR of 10.08 ± 5.36 months (range, 3.6–23.6 months). Patients No. 6 and No. 13 were diagnosed with adenomyosis, whereas none of the patients presented with either fibromatosis or adnexal pathologies. Seven out of sixteen patients (43.8%) remained in remission for more than 9 months. IVF treatments were administered after a median duration of 3.45 months (range, 0.97–14.43 months) after CR. Subsequent FETs were performed after a mean duration of 5.24 ± 2.51 months (range, 2.07–11.23 months) after the last COS cycle. A total of 26 COS cycles were performed on the sixteen patients, with a mean number of 1.63 ± 0.62 COS cycles per patient. Ovarian stimulation lasted for a mean duration of 10.54 ± 2.89 days (range, 5–17 days), with a mean estradiol level of 4723.85 ± 2650.33 pmol/L (range, 248–9357 pmol/L) on the day of human chorionic gonadotropin (hCG) triggering. The mean number of oocytes retrieved per cycle was 9.81 ± 5.59 (range, 2–23).

**Table 1:** Clinical findings and follow-up information of the analysis cohort

Case	Age, y	BMI, kg/m^2^	Histological type	Time to CR (mo.)	Time to COS (mo.)	Time to first ET (mo.)	No. of COSs	No. of ETs	COS to Relapse(mo.)	Pregnancy outcomes	Time to LB (mo.)	Follow up, mo./status
1	32	30.0	G2	23.6	6.0	6.8	2	1	–	–	–	23.80/NED
2	31	25.4	G1	3.6	12.7	8.4	1	1	–	–	–	30.57/NED
3	30	27.6	G1	5.7	3.0	2.1	1	2	–	LB (35^th^ wk.)	12.03	21.40/NED
4	34	26.2	G1	7.7	3.8	0	1	0	–	–	–	24.17/NED
5	38	32.0	G2	7.3	3.2	0	2	0	16.23	–	–	20.20/Under retreatment
6	35	24.6	G1	9.5	3.7	0	1	0	–	–	–	21.63/NED
7	35	31.5	G1	7.3	4.2	4.5	2	1	–	–	–	20.97/NED
8	27	28.0	G1	7.2	5.1	3.8	1	2	–	LB (41^st^ wk.)	15.23	32.03/NED
9	38	22.8	G1	19.3	14.4	4.1	2	2	11.67	–	–	42.30/Hysterectomy, NED
10	32	26.6	G1	11.8	2.0	3.4	2	1	–	LB (38^th^ wk.)	13.63	23.53/NED
11	37	30.2	G1	6.9	1.1	3.7	2	2	–	M (16^th^ wk.) LB (37^th^ wk.)	27.3	28.40/NED
12	30	21.3	G1	8.5	1.0	0	2	0	–	LB (38^th^ wk.) *	15.87*	24.63/NED
13	25	37.9	G1	9.7	3.1	5.1	2	1	–	LB (39^th^ wk.)	16.67	30.13/NED
14	32	24.8	G1	6.0	6.8	11	1	2	–	EP	–	79.80/NED
15	36	28.0	G2	16.3	3.2	4.1	1	1	6.63	–	–	55.47/ Under retreatment
16	39	32.3	G1	10.8	2.9	5.7	3	3	–	M (6^th^ wk.)	–	22/NED

*Spontaneous pregnancy, *EEC* endometrioid endometrial cancer, *BMI* body mass index, *CR* complete response, *COS* controlled ovarian stimulation, *ET* embryo transfer, *NED* no evidence of disease, *LB* live birth, *EP* ectopic pregnancy, *M* miscarriage

**Table 2:** Characteristics of COS and outcomes of pregnancy

Subject characteristic	*n* = 16
Age (y), mean ± SD	33.19 ± 4.04
BMI (kg/m^2^), mean ± SD	28.07 ± 4.16
No. of D&Cs, mean ± SD	5 ± 0.97
Basal FSH (U/L), mean ± SD	5.84 ± 2.95
AMH (ng/mL), median (range)	1.55 (0.38–10.0)
Treatment protocols, *n* (%)	
Oral progestin	12 (75.0)
LNG-IUD + Oral progestin/ GnRH agonist	4 (25.0)
No. of COS cycles, *n*	26
GnRH agonist, *n* (%)	9 (34.6)
GnRH antagonist, *n* (%)	10 (38.5)
Letrozole + GnRH antagonist/agonist, *n* (%)	7 (26.9)
Total dose of Gn (IU), mean ± SD	2867.31 ± 1252.09
E_2_ on hCG day (pmol/L), mean ± SD	4723.85 ± 2650.33
No. of oocytes retrieved, mean ± SD	9.81 ± 5.59
No. of available embryos, mean ± SD	3.73 ± 2.25
No. of ETs, *n*	19
No. of natural cycle, *n* (%)	6 (31.6)
No. of induced cycle, *n* (%)	5 (26.3)
No. of artificial cycle, *n* (%)	8 (42.1)
No. of embryos transferred per ET, mean ± SD	1.63 ± 0.50
Clinical pregnancy, *n* (%)	7/19 (36.8)
Live birth, *n* (%)	5/19 (26.3)
Miscarriage, *n* (%)	2/19 (10.5)

*COS* controlled ovarian stimulation, *SD* standard deviation, *AMH* anti-müllerian hormone, *D&C* dilation and curettage, *LNG-IUD* levonorgestrel-releasing intrauterine device, *Gn* gonadotropin, *hCG* human chorionic gonadotropin, *E*_*2*_ estradiol, *ET* embryo transfer

Twelve patients underwent 19 subsequent thaw embryo transfer cycles, with a mean number of 1.19 ± 0.91 ET cycles (range, 1–3 ET cycles) and a mean number of 1.63 ± 0.50 (range 1–2) embryos transferred. Five patients had FET performed with artificial cycles: Patients No. 3, No. 10, and No.8 due to ovulation disorders, and Patients No.2 and 16 because of a thin endometrium (endometrium thickness before luteal support ≤ 7 mm). None of these patients experienced a relapse. Out of 19 cryopreserved ET cycles, 10 (32.26%) of 31 embryos were implanted, resulting in 1 ectopic pregnancy, 2 miscarriages and 5 live births. The clinical pregnancy and live birth rates were 36.8% (7/19) and 26.3% (5/19), respectively, per FET cycle. Five patients gave birth to 6 healthy live neonates. Among the two patients with miscarriage, one (No.11) had a miscarriage at 16 weeks of gestation with twin pregnancies due to cervical insufficiency and subsequent live birth was achieved by the second FET. The other miscarriage occurred in 6^th^ week (No.16). Seven out of 16 participants were over the age of 35 and only one achieved a successful live birth. Out of the 16 patients in the study, four patients were unable to proceed with embryo transfer for various reasons. No. 5 got disease recurrence during the treatment with estradiol for thin endometrium, No. 6 did not obtain transferable embryos, No. 12 experienced spontaneous pregnancy with term delivery before embryo transfer, and No. 4 postponed FET with maintenance of LNG-IUD for personal reason. Therefore, the cumulative pregnancy and live birth rate during the study period were 43.75% (7/16) and 37.5.% (6/16), respectively.

Seven out of 16 patients were diagnosed with thin endometrium (No. 2, No. 5, No. 6, No. 7, No. 13, No. 15, and No. 16). Among them, five patients underwent a total of seven ET cycles (No. 2, No. 7, No. 13, No. 15, and No. 16), and only No. 13 achieved a live birth born at 39th week. Furthermore, during the treatment with estradiol for thin endometrium, two patients (No. 5 and No. 15) experienced disease relapse.

The mean follow-up time was 31.31 ± 15.89 months (range, 20.2–79.80). During the follow-up period, three patients (No. 5, 9, and 15) experienced recurrence after COS. The time intervals from COS to relapse were 16.23, 11.67 and 6.63 months, respectively. After two consecutive COS cycles with letrozole/GnRH agonist protocol and letrozole/GnRH antagonist protocol in Patient No.5, the LNG-IUD was removed. During the treatment with estradiol for thin endometrium and intrauterine adhesion, the FET was cancelled and followed by disease recurrence. The patient was subsequently re-treated with MPA until the end of the follow-up period. Patient No. 9 received two COS cycles with GnRH antagonist protocols, two ET cycles were performed with frozen-thawed cleavage stage embryos transferred while the endometrium were 9 mm. Unfortunately, she did not achieve pregnancy. Three months after the last FET, the patient underwent hysterectomy and superficial myometrial invasion was confirmed by postoperative pathological results. Patient No. 15 had one COS cycle with GnRH antagonist protocol, followed by one FET with a 5 mm endometrium and implantation failure. She experienced a relapse during treatment for the thin endometrium and is currently undergoing retreatment with MA, letrozole, and metformin.

Three patients (No. 1, No. 5, and No. 15) were diagnosed with grade 2 EEC. Patients No. 5 and No. 15 underwent one FET cycle each, but neither resulted in a pregnancy. Both patients experienced a relapse, with the duration between their last COS and relapse being 16.23 and 6.63 months, respectively. Patient No.1 underwent two cycles of COS followed by one cycle of FET with a 10 mm endometrium, however, did not achieve pregnancy.

## Discussion

LNG-IUD has a distinct progesterone effect on endometrium, opposing the action of estrogens. LNG-IUD-based therapies are increasingly being adopted as promising conservative alternatives for managing ECC patients [13, 14]. As shown in the previous study, EEC patients were more likely to relapse, with a recurrence rate of 27.27–50% after COS [8, 10]. In the current study, 18.75% (3/16) of EEC patients who underwent COS with LNG-IUDs inserted experienced a relapse, with a follow-up time of 31.31 ± 15.89 months. Among these three relapse cases, two of them were initially diagnosed with moderately-differentiated EEC and relapse lesions still localized in the endometrium. A recent meta-analysis (2022) of 84 patients with grade 2 endometrial cancer revealed that 54 (64.3%) achieved a complete response, 22 (26.19%) accomplished pregnancy, while 20 (23.8%) experienced a recurrence [15]. Possibly due to the lower response rate of poorly differentiated endometrial cancer to progestogens than highly differentiated ones and a higher risk of recurrence, the current guidelines do not indicate fertility-sparing treatment for early-stage moderately differentiated endometrial cancer [16]. The reported data on this topic are rather limited, patients must be fully informed about the benefits and risks associated with this treatment protocol, especially possibilities of recurrence and the importance of follow-up. With the introduction of the new endometrial cancer staging system in 2023, incorporating molecular subtyping, a more refined classification of patients has been achieved. This advancement would facilitate the development of tailored treatment approaches targeting various risk factors.

The E_2_ level on hCG trigger day and the duration of Gn consumption may be related to the relapse of endometrial diseases [17]. The use of letrozole, a highly selective non-steroidal oral aromatase inhibitor, has been reported to result in lower levels of estradiol compared to the standard stimulation cycle [18] and it may stifle the growth of uterine endometrial cancer [19]. Although there may be fewer oocytes retrieved and a high cycle cancellation rate with the use of letrozole protocols in both the general IVF population and poor responders, the clinical pregnancy and live birth rates remain unclear[20].

Repeated curettage in patients with EEC may inflict irreversible mechanical damage to the endometrium, potentially causing endometritis, intrauterine adhesion, and endometrial thinning, which negatively impacts endometrial receptivity [21]. LNG-IUD combined with hysteroscopic endometrium biopsy possibly decrease the chance of injury to endometrium. A natural or stimulated ovulation cycle FET is strongly recommended in EEC patients. However, women with thin endometrium might be recommended with the artificial cycle to promote endometrial growth. The risk of increased estrogen levels on tumor recurrence should be a concerned and the balance of effectiveness and risks should be evaluated regularly. In this study, artificial cycles were used in two patients (No. 2 and No.16) with thin endometrium and the endometrium did not improve. Reducing treatment duration, enhancing treatment efficacy, and minimizing intrauterine procedures may help to decrease endometrial damage and potentially improve pregnancy outcomes. Various potential treatments for thin endometrium, such as high-dose estrogen replacement therapy, growth hormone, aspirin, sildenafil, uterine perfusion, and colony-stimulating factor, are currently being explored. However, there's still a lack of research regarding whether these treatments increase the risk of endometrial tumor recurrence, necessitating further studies.

Current evidence suggests that maintaining an LNG-IUD during COS might reduce disease relapse without negatively impacting subsequent pregnancies in women with EEC. Adeleye et.al[9]reported that there was no significant difference in oocyte yield and mature oocyte yield between subjects with and without an LNG-IUD in women pursing social oocyte cryopreservation and oocyte donors. The predicted clinical pregnancy rate and live birth rate did not differ significantly for oocyte recipients. LNG-IUD can be applied for patients who require multiple ovulation stimulations to accumulate embryos for future pregnancy. Furthermore, for patients who desire fertility cryopreservation, maintaining an LNG-IUD during COS may serve as a safer strategy for preserving the endometrium. Vigilance is still required as there is a lack of high-quality evidence-based medical research. The LNG released by LNG-IUD might not completely antagonize the recurrence of endometrial lesions, hence refined surveillance strategies are required. Molecular classification has the potential to improve risk stratification when integrated with clinicopathologic features and further studies are needed to provide more information to guide treatment strategy decision. Patient education is quite crucial. Patients should be fully informed about the benefits associated with this treatment protocol, potential side effects, likelihood of success, and possibilities of recurrence to ensure that they can make informed decisions about their care and understand the importance of adhering to the prescribed treatment plan and regular follow-ups. The present study has limitations related to the retrospective study design and the small sample size. More multicenter prospective studies need to be performed.

## Conclusions

The combination of LNG-IUD maintenance treatment and segmented IVF might be a viable and endometrium-protective therapy for stimulating ovulation during IVF in patients conservatively treated for early-stage EEC, especially for EEC patients who desire to cryopreserve fertility.
